# A Unique Association of Osteogenesis Imperfecta with Bilateral Renal Osteodystrophy and Gastroenteritis in a Three-year-old Boy

**DOI:** 10.7759/cureus.4467

**Published:** 2019-04-16

**Authors:** Laila Tul Qadar, Rohan Kumar Ochani, Asim Shaikh, Qazi Arsalan, Ramsha Ali

**Affiliations:** 1 Internal Medicine, Dow University of Health Sciences, Karachi, PAK; 2 Pediatrics, Dow University of Health Sciences, Karachi, PAK

**Keywords:** osteogenesis imperfecta, brittle bone disease, collagen, mutation

## Abstract

We describe a three-year-old male child who presented to the pediatrics out-patient department with a history of decrease in appetite, generalized weakness, on and off loose motions for one year, inability to walk and sit for eight months with a loss of neck holding for 14 days. On examination, the patient had a classic frog-shaped leg posture. X-rays of chest, skull, pelvis and long bones were performed which showed osteopenic bones, frontal bossing and multiple microfractures, which were classic for osteogenesis Imperfecta but the child did not have other salient features such as blue sclera, otosclerosis, and respiratory difficulty. The patient also had urinary complaints due to which ultrasound of kidney ureters and bladder (KUB) was performed, which showed bilateral renal calculi and grade 2 renal parenchymal changes. This case report illustrates the evaluation of the child with osteogenesis imperfecta, as well as the unique association of renal osteodystrophy and gastroenteritis with it.

## Introduction

Osteogenesis imperfecta (OI), also known as brittle bone disease, is a group of inherited connective tissue disorders, which happens due to mutations that affect collagen in the connective tissue of the body resulting in fragile bones. Therefore, people with this condition have bones that break easily, often due to a mild trauma or with no apparent cause. There are eight recognized forms of osteogenesis imperfecta, type I to type VIII. The types can be differentiated by their clinical presentations, although, some of their characteristic features overlap. Type I is the mildest form, while type II is the most severe form and the remaining types have signs and symptoms that range between these two extremes [1].

Collagen is the major protein of the body’s connective tissue and plays an essential role in building strong bones. OI is caused by genetic defects that mainly affects collagen synthesis or quality of collagen resulting in weak, fragile bones. Most cases of OI have an autosomal dominant pattern of inheritance, which is caused by a mutation in one of the type 1 collagen genes (COL1A1 and COL1A2); therefore, a person has either too little type 1 collagen or poor quality of type 1 collagen. In recessive OI, the mutation does not affect the main collagen genes and results from mutations in other genes which interfere with the collagen production. The result in all cases is weak bones that break easily [2]. Diagnosis is often based on symptoms and may be confirmed by collagen or DNA testing [3].

## Case presentation

A three-year-old male child was admitted to the pediatric ward of Dr. Ruth KM Pfau, Civil Hospital Karachi (CHK) with a one-year history of generalized weakness, loose motions, decreased appetite and intermittent fever not associated with rigors, chills or night sweats, an eight-month history of inability to walk and sit, polydipsia, polyurea, abdominal distention and loss of neck holding for 14 days. He had a previous history of hospitalization six months ago due to the same complaints. The patient was accompanied by his mother. He weighed 6 kg, is the 7th born child to his parents and was delivered at term to a 37-year-old G7P7 mother via normal vaginal delivery. The mother did not report any complications or illnesses during pregnancy. He cried immediately after birth, and there were no complications during or after birth. There is no consanguinity between mother and father. The child was vaccinated but was malnourished, with an unremarkable family history.

The patient also developed diarrhea which was bulky in consistency, green in color with seven episodes per day after every meal intake. Diarrhea was associated with abdominal distention and vomiting. The mother then started giving him a combination of trimethoprim-sulfamethoxazole, after which diarrhea subsided. The child developed neck holding at four years of age, he started sitting at eight months and started walking with support at 12 months of age. The child was first breastfed within three hours of delivery and was exclusively breastfed up to six months. Complimentary feeding was started after six months with pulses, mashed potatoes, porridge and chicken. Breastfeeding was continued up to two years. Currently, the child takes 1-2 feeds per day. The personal history revealed that the patient was sleeping normally, but the appetite was decreased. The mother also noticed a weight loss, altered bowel habits, and micturition was normal.

**Figure 1:**
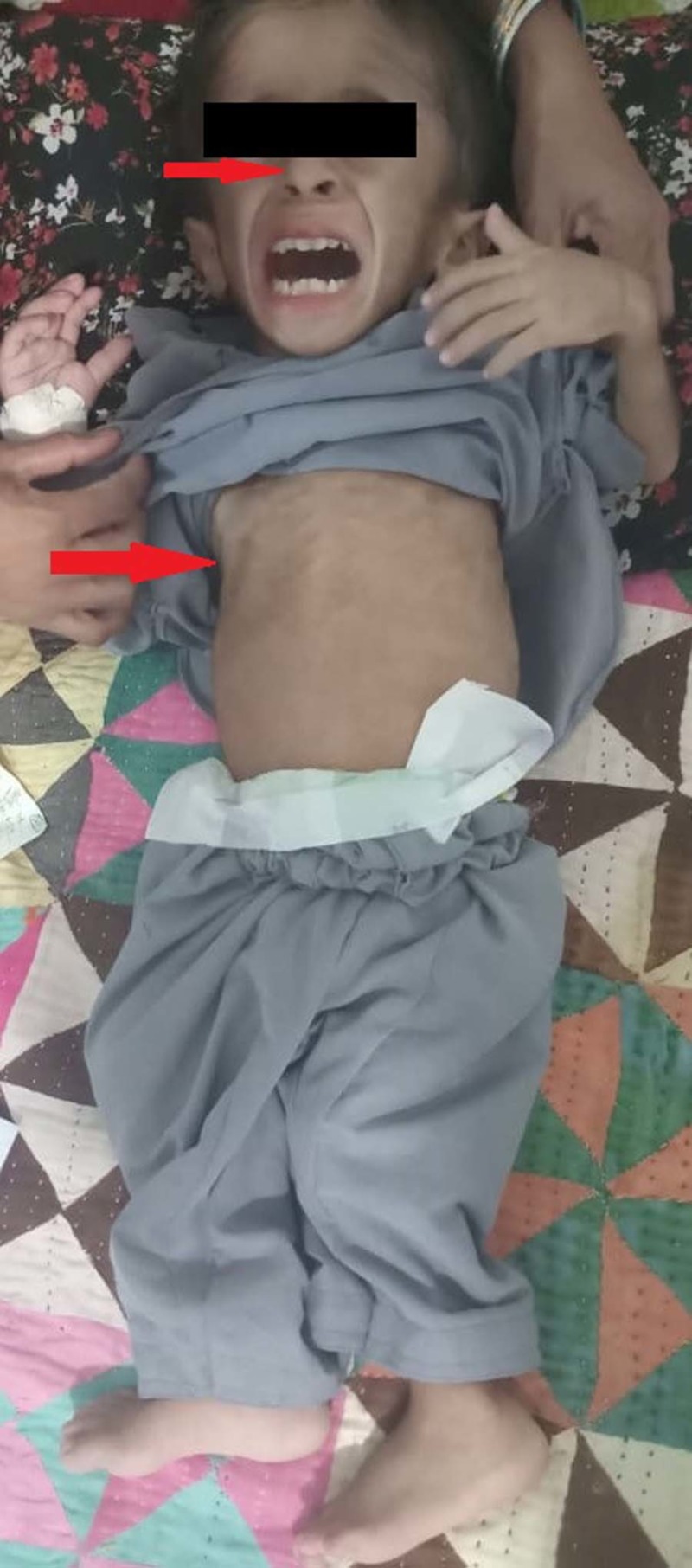
OI patient severely wasted and emaciated with visible bony deformities, depicting parietal and frontal bossing and rachitic rosary. OI: Osteogenesis imperfecta

On examination (O/E), the patient was lying on the bed irritated, severely wasted and emaciated with visible bony deformities. Initial vitals included blood pressure (BP) 110/70 mmHg, a regular pulse of 90 beats/min, a respiratory rate of 20 breaths/min, and a low-grade fever of 100° F. The patient was anemic and dehydrated, while, there was no presence of edema, clubbing, cyanosis, and lymphadenopathy. On further examination, parietal and frontal bossing, rachitic rosary (Figure 1), widening of wrists and knock knees (it's a valgus deformity in which legs curve inwards so that the feet are apart when the knees are touching) were found as well. On abdominal examination, it was soft, non-tender, distended with mild tenderness on both flank regions, with a centrally placed umbilicus. The liver was palpable three fingers below the right costal margin. Gut sounds were audible 3-4 sounds/min. All other systems were unremarkable.

The anthropometric measures of the child are as follows: weight = 6.5 kg, occipitofrontal circumference = 49.5 cm, height = 72.5 cm, mid-upper arm circumference = 8 cm, upper segment = 45 cm, lower segment = 27.5 cm, weight for height = 6.5/9 *100 = 72.2 (moderately low) and height for age = 72.5/94.6*100 = 76.6 (severely low).

On investigations performed, complete blood count (CBC) showed a hemoglobin (Hb) of 5.4 gm/dl, mean corpuscular volume (MCV) of 82 fl and a platelet count of 187,000/mL. The total leukocyte count (TLC) was 10.6 x 10^9^/L, including 39% neutrophils and 48% lymphocytes. His inflammatory markers were raised with a C-reactive protein (CRP) of 23 mg/L [Normal (N) = 3] and an elevated erythrocyte sedimentation rate (ESR) of 115 mm/hr (N = 0-22) for men and (N = 0-29) for women. The clotting profile showed an international normalized ratio (INR) of 1.01, while prothrombin time (PT) was 10.6 seconds.

The urea creatinine electrolytes (UCE) were within normal range except decreased potassium levels of 2.8 mEq/L. The levels of calcium, magnesium and phosphate in blood were 15.1, 1.9 and 4 mg/dL, respectively. The blood culture showed a growth of Burkholderia species. The urinalysis showed a pH of 5.0, specific gravity of 1.025, with the presence of protein and blood. Additionally, numerous red cells were seen along with yeast and 4-6 pus cells per high power field (HPF). The urine culture showed growth of Candida species. The stool analysis showed that the stool was hard, acidic, brown in color with 1-2 pus cells per HPF. His vitamin D levels came out to be 54.29 ng/mL, and parathyroid levels were 8.36 pg/ml. Liver function tests showed a total bilirubin of 0.28 mg/dL, direct bilirubin of 0.21 mg/dL and levels of alkaline phosphate alanine aminotransferase were 368 and 3 units per liter, respectively.

**Figure 2:**
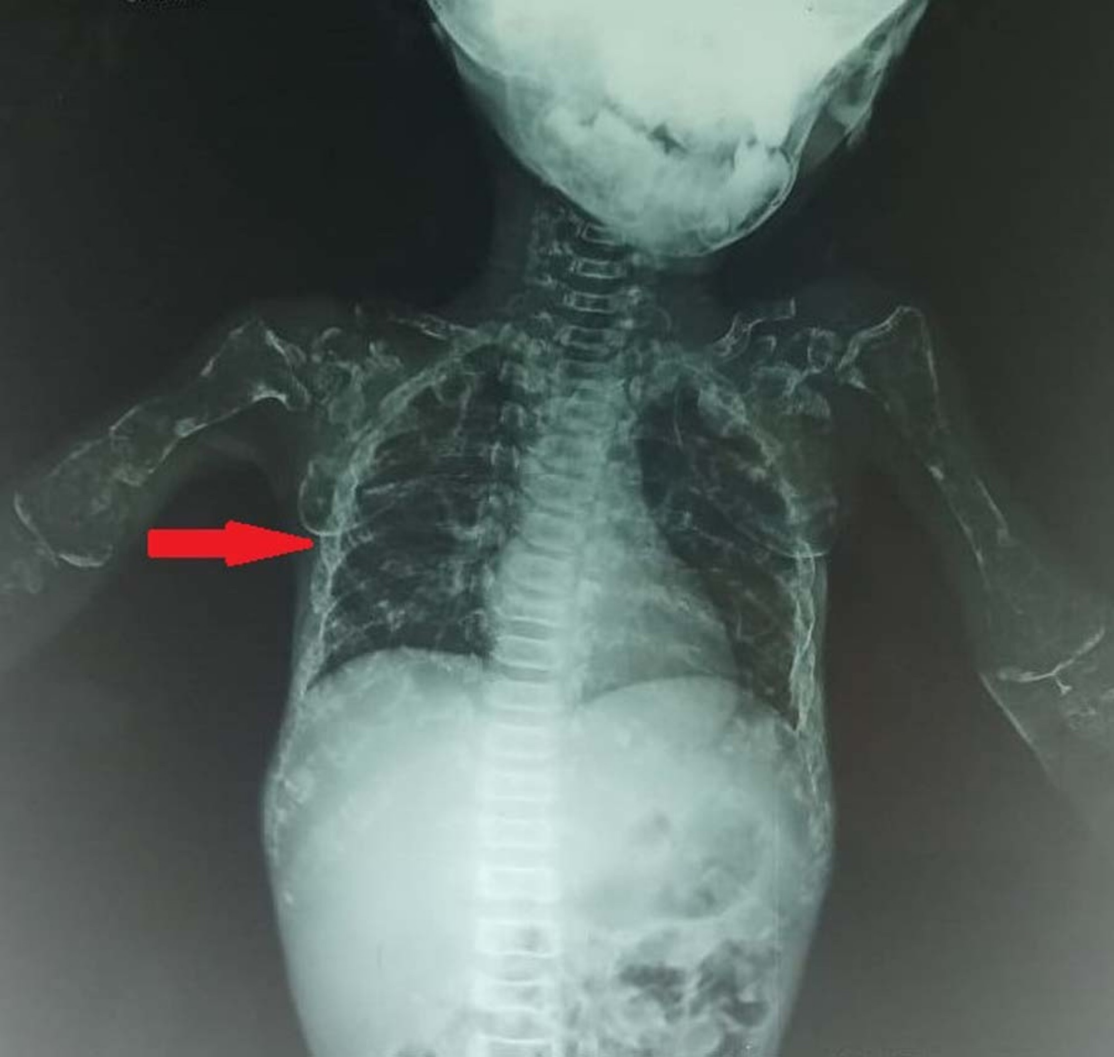
Chest X-ray showing excessive amount of multiple rib fractures.

**Figure 3:**
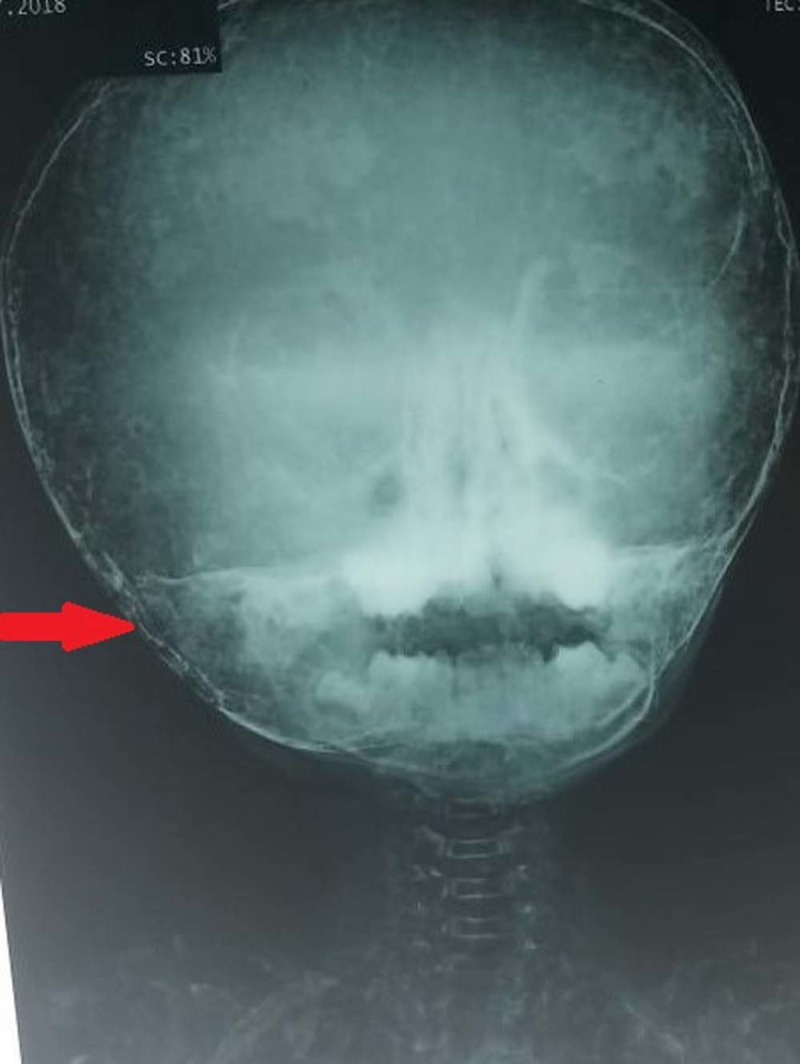
Skull X-ray showing thinning of the skull with multiple ossification centres.

**Figure 4:**
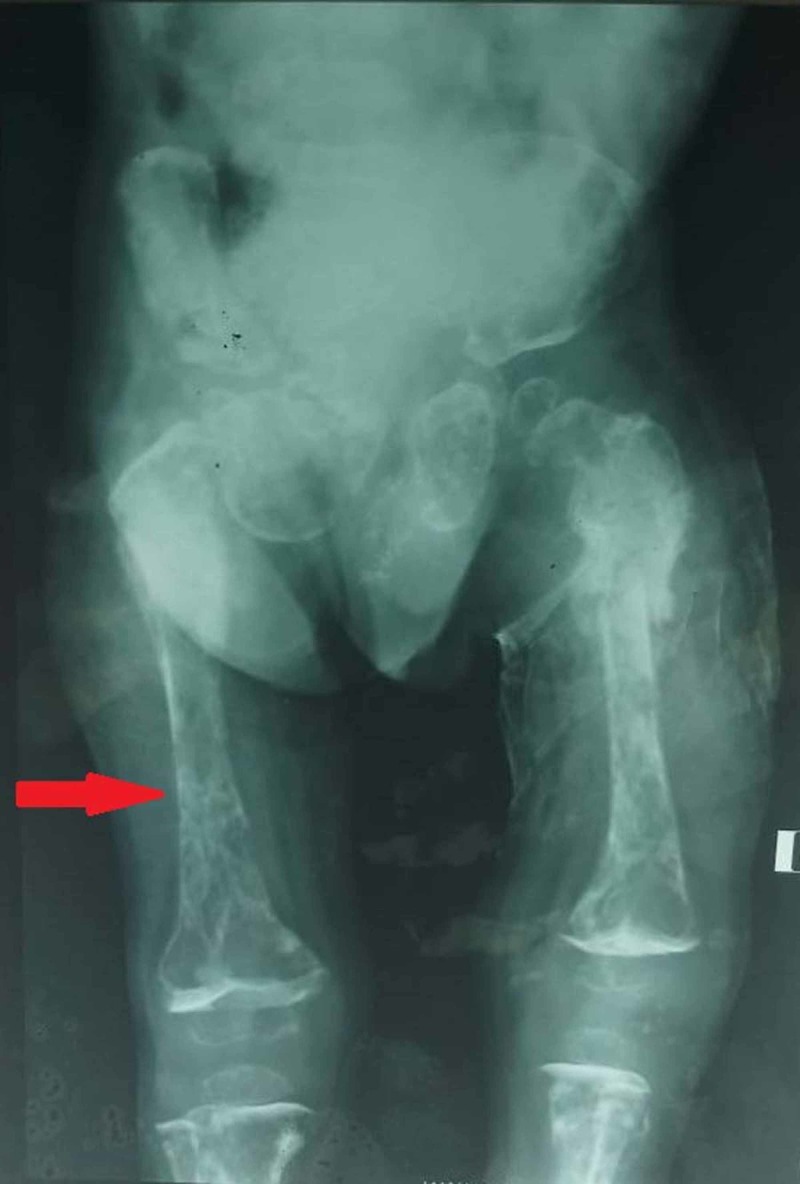
Femur X-ray of OI patient showing multiple femur fractures. OI: Osteogenesis imperfecta

The X-rays of the chest (Figure 2), skull (Figure 3) and femur (Figure 4) are attached below. Additionally, ultrasound of kidney ureters and bladder (KUB) showed an incidental finding of bilateral renal calculi (right kidney at lower pole measuring 0.7 cm, in the left kidney at mid pole measuring 0.7 cm), along with bilateral grade 2 renal parenchymal changes. The urinary bladder was normal in thickness with no presence of focal mass, calculus or diverticulum.

Considering the diagnosis of OI, an ophthalmology review was done to look for lens dislocation and blue sclera, but neither was present in both eyes. However, the left eye showed a corneal thinning nasally, temporally and inferiorly.

During the hospital stay, the patient was intravenously given cefotaxime 220 mg, piperacillin-tazobactam 700 mg, and meropenem three times a day. Additionally, linezolid 70 mg was injected every eight hours, along with injections of bisphosphonate 7 mg for three days, amikacin 55 mg twice a day for 14 days and fluconazole 85 mg loading dose, 45 mg once a day for 10 days. The patient was also given a teaspoon of the combination (artemether and lumefantrine) once a day. Three pints of red blood cells were transfused as well, after which his Hb became 10.6 mg/dL. During the stay, the child had a prolonged course of fever with pancytopenia, which improved on injections of meropenem, amikacin, and fluconazole.

## Discussion

OI is a genetic disorder characterized by a varying group of diseases. The hallmark of OI is an increased vulnerability to fractures ranging from slight to pre-natal. Other common features include a distinct blue sclera, hyper-elastic skin and ligaments, impaired hearing, bone deformities, short statures and dentinogenesis imperfecta (DI) [4]. With an incidence of 1 in 10,000-20,000 births, it also results in abnormal blood coagulation, airway obstruction and cardiovascular anomalies [4].

OI was designated a widely accepted classification in 1979 by Seillence et al. The disorder was classified into four types depending on symptoms and presentation: Type I; blue sclera and dominant inheritance, Type II; perinatally lethal with crumpled femora, Type III; progressively deforming and no discoloration of the sclera and Type IV; no discoloration of sclera with dominant inheritance. Type I and II defer in that the former is mild and has two subtypes, A and B, depending on the presence of DI while the latter is lethal and inherited autosomal recessively. Type III and IV have intermediate presentations with Type III being the most severe form that is non-lethal while Type IV also has two subtypes similar to Type I. Since then, five more types have been classified depending on histological findings [5].

The various phenotypic variations all result from mutations in one of two genes, COL1A1 and COL1A2 which encode for pro-α1(I) or pro-α2(I) chains of type I collagen [5] which results in defective qualitative or quantitative integrity of type I collagen, the principal protein in bones and the extracellular matrix [4].

In the present case, the diagnosis was made based on the multiple micro-fractures, frontal bossing and the osteopenic bones exhibited by the patient during X-ray imaging. While such a presentation is classic for OI, the presence of multiple other features such as blue sclera, perinatal lethality, and respiratory problems makes diagnosis and classification easier, which were not noted in this case. Physical examination only revealed an emaciated, malnourished child with visible bony deformities without the presence of DI or other classically associated features but parietal and frontal bossing, Rachitic rosary, widening of wrists and knock knees were observed which aided the diagnosis [6,7]. The patient also had a fever and diarrhea.

Furthermore, the most unique feature of this case, besides the lack of other OI identifying associations, was what ultrasound KUB revealed. We observed that the patient had renal calculi and grade II renal parenchymal changes. While chronic renal failure from renal artery occlusion in OI owing to pelvic deformities has been rarely documented [8], the presence of renal calculi has been even more rarely reported. A study conducted in 1989 suggested that renal calculi might be a manifestation of OI, but cardiac anomalies were more common [9]. Therefore, renal manifestations are perhaps a crucial extra musculoskeletal feature of OI.

The treatment regimen involved administration of multiple antibiotics, antifungals, and antimalarials to treat the infection, fever, and diarrhea as it presented a more immediate threat followed by blood transfusions [10].

While OI results in a multitude of complications, to date there is no surgery or known treatment that will effectively treat and cure OI. The mainstay of therapy is care of fractures, physiotherapy and the use of bisphosphonates to maintain bone mineral density by inhibiting osteoclastic activity and therefore reducing bone resorption [11] and treating other symptoms such as hearing loss with hearing aids. There are numerous therapeutic strategies which are designed to help afflicted individuals attain a normal life as identified by various studies [12,13].

The therapeutic goal we developed was based on curing the infection and diarrhea and anemia followed by helping to manage the renal parenchymal disease found in the patient and helping to manage the osteopenia demonstrated.

While the treatment therapies mentioned previously form the current gold standard of OI management, new pharmacological interventions such as antibodies to sclerostin, mesenchymal stem cell transplantation, and gene therapy with specific allele silencing [14], might prove to be much more effective in combating and perhaps eradicating OI.

## Conclusions

The present case report, in conjunction with previous literature, albeit scarce, suggests that renal manifestations of OI need to be considered as a grave complication or association. While the renal parenchymal disease has been documented to some extent, the presence of renal calculi is a perhaps novel finding in OI patients. Therefore, we recommend that suspecting renal disease in patients of OI should be suspected and physicians should be aware of its possible existence. Complete workup for the renal function should be carried out, and preemptive strategies to combat chronic renal failure in OI should be specified and fine-tuned.

## References

[1] (2019). Osteogenesis imperfecta. https://web.archive.org/web/20161018224156/https://ghr.nlm.nih.gov/condition/osteogenesis-imperfecta.

[2] (2019). Osteogenesis imperfecta overview. https://www.bones.nih.gov/health-info/bone/osteogenesis-imperfecta/overview.

[3] Byers PH, Krakow D, Nunes ME, Pepin M (2006). Genetic evaluation of suspected osteogenesis imperfecta (OI). Genet Med.

[4] Ren J, Xu X, Jian X, Wang J (2014). Osteogenesis imperfecta type I: a case report. Exp Ther Med.

[5] Edelu B, Ndu I, Asinobi I, Obu H, Adimora G (2014). Osteogenesis imperfecta: a case report and review of literature. Ann Med Health Sci Res.

[6] (2019). Rachitic rosary. https://www.ncbi.nlm.nih.gov/medgen/140878.

[7] Heide T (1981). A syndrome of osteogenesis imperfecta, macrocephaly, wormian bones, frontal bossing, brachytelephalangy, hyperextensible joints, congenital blindness and oligophrenia in 3 sibs (author's transl). (Article in German). Klin Padiatr.

[8] Butani L, Rosekrans JA, Morgenstern BZ, Milliner DS (1995). An unusual renal complication in a patient with osteogenesis imperfecta. Am J Kidney Dis.

[9] Vetter U, Maierhofer B, Müller M (1989). Osteogenesis imperfecta in childhood: cardiac and renal manifestations. Eur J Pediatr.

[10] Koletzko S, Osterrieder S (2009). Acute infectious diarrhea in children. Dtsch Arztebl Int.

[11] Drake MT, Clarke BL, Khosla S (2008). Bisphosphonates: mechanism of action and role in clinical practice. Mayo Clin Proc.

[12] Monti E, Mottes M, Fraschini P (2010). Current and emerging treatments for the management of osteogenesis imperfecta. Ther Clin Risk Manag.

[13] Devogelaer JP, Coppin C (2006). Osteogenesis imperfecta: current treatment options and future prospects. Treat Endocrinol.

[14] Shaker JL, Albert C, Fritz J, Harris G (2015). Recent developments in osteogenesis imperfecta [version 1; peer review: 3 approved]. F1000Res.

